# Architectural plasticity of AMPK revealed by electron microscopy and X-ray crystallography

**DOI:** 10.1038/srep24191

**Published:** 2016-04-11

**Authors:** Yan Ouyang, Li Zhu, Yifang Li, Miaomiao Guo, Yang Liu, Jin Cheng, Jing Zhao, Yi Wu

**Affiliations:** 1 School of Life Sciences, Lanzhou University, Lanzhou 730000, China

## Abstract

Mammalian AMP-activated protein kinase (AMPK) acts as an important sensor of cellular energy homeostasis related with AMP/ADP to ATP ratio. The overall architecture of AMPK has been determined in either homotrimer or monomer form by electron microscopy (EM) and X-ray crystallography successively. Accordingly proposed models have consistently revealed a key role of the α subunit linker in sensing adenosine nucleoside binding on the γ subunit and mediating allosteric regulation of kinase domain (KD) activity, whereas there are vital differences in orienting N-terminus of α subunit and locating carbohydrate-binding module (CBM) of β subunit. Given that Mg^2+^, an indispensable cofactor of AMPK was present in the EM sample preparation buffer however absent when forming crystals, here we carried out further reconstructions without Mg^2+^ to expectably inspect if this ion may contribute to this difference. However, no essential alteration has been found in this study compared to our early work. Further analyses indicate that the intra-molecular movement of the KD and CBM are most likely due to the flexible linkage of the disordered linkers with the rest portion as well as a contribution from the plasticity in the inter-molecular assembly mode, which might ulteriorly reveal an architectural complication of AMPK.

AMP-activated protein kinase (AMPK), an αβγ heterotrimeric complex, plays an essential role in regulating cellular metabolism and maintaining energy homeostasis by sensing AMP/ADP to ATP ratio in mammals^1^,^2^,^3^. The γ subunit contains four tandem repeats of a cystathionine-β-synthase domain (CBS1–4) for adenine nucleotide binding except site 2 (CBS-2). The α subunit comprises an N-terminal kinase domain (KD), followed by the regulatory region encompassing an autoinhibitory domain (AID) and a regulatory linker region (α-linker) which included two tandem α regulatory-subunit-interacting motifs (α-RIMs), of which the RIM-1 binds to the unoccupied CBS-2 site^4^,^5^ while the RIM-2 (originally referred to as α-hook) has been affirmed to directly interact with CBS-3 site on γ subunit in mediating the allosteric regulation of AMPK activity^4^,^5^,^6^,^7^. The KD can be potently inhibited by compound staurosporine^8^,^9^. Besides an unstructured portion at the beginning, the N-terminus of β subunit consists of a carbohydrate binding module (CBM, also termed GBD) which can bind glycogen and small molecular activators^9^,^10^,^11^,^12^,^13^. The C-terminal domains of α and β subunits (termed as α-CTD and β-CTD respectively) together with the entire γ subunit constitute the regulatory core complex, which occupies almost 50% volume of the whole structure.

In the past five years, several groups have reported the overall architecture of mammalian AMPK in either homotrimer or monomer form by electron microscopy (EM) and X-ray crystallography successively. Those structures were determined in varied functional preparations, primarily focusing on the threonine 172 (rat α1, human α2) within the activation loop of KD, whose phosphorylation by the upstream kinases^14^,^15^,^16^,^17^,^18^,^19^ defines the prerequisite of an active enzyme, the CBM and its activators, and binding of AMP/ADP to the CBS-3 site and CBS-4 site of γ subunit for an allosteric modulation or protecting α-Thr172 against dephosphorylation^5^,^9^,^20^,^21^. They represent almost consistent characteristics in the relative location of KD to the regulation core complex, orienting AID related to KD, and discovering α-RIMs in the allosteric activation. However, there’re notable architectural differences in between structures resolved by EM and crystallography about orienting KD-AID to the regulatory core complex as well as the location of CBM.

Considering that Mg^2+^, an essential cofactor of protein kinase no exception for AMPK^22^,^23^,^24^,^25^, was contained in our previous working buffer but not added in crystalline hanging drops, here we performed further reconstructions for AMPK without Mg^2+^ by single-particle electron microscopy to eliminate the possibility caused by this ion. It turns out that AMPK is able to form similar homotrimer in Mg^2+^-free buffer in both vacant and AMP binding states, and this oligomeric form can be influenced by pH and salt concentration, which implies that the electrostatic interaction between individual protomer would participate in mediating AMPK oligomerization. No significant difference observed between the EM models of unphosphorylated AMPK generated in the present and absent of Mg^2+^ suggests that this divalent cation do not alter the architecture of AMPK in the EM experimental system. Further analyses have revealed the disordered linker sequences together with the intra- and inter-molecular interactions may contribute to a certain plasticity of AMPK architecture, which in turn demonstrates an architectural complication of this enzyme complex.

## Results

### AMPK homotrimer formed in Mg^2+^-free buffer

**Figure 1:**
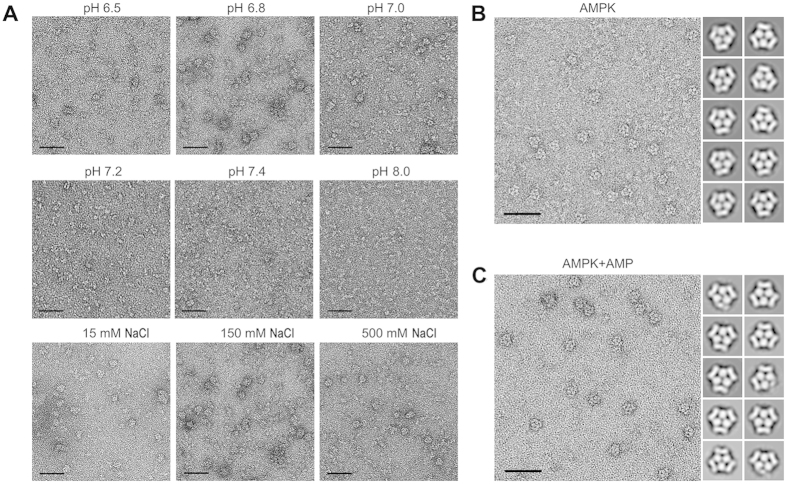
Observation of AMPK by negative staining. (**A**) Typical EM micrographs of AMPK prepared in buffers with 150mM NaCl but different pH (top and middle rows) and with a fixed pH of 6.8 but different salt concentration (bottom row). Typical EM micrograph prepared in Mg^2+^-free buffer and related 2D class average map of AMPK (**B**) and AMPK incubated with 1mM AMP (**C**), respectively. Scale bar = 50 nm.

In the previous work, we prepared AMPK in Mg^2+^-containing buffer (1.2 mM MgCl_2_) and obtained its three-dimensional structure in homotrimeric form by RCT method^20^. When we excluded Mg^2+^ in this study, AMPK represents the same ability to form homotrimer as well, appearing similar triangular shape with repeated globular and rod-like density. Additionally, AMPK were examined after diluted in buffers with gradually varied pH and different salt concentration (Fig. 1A). The observation results showed that AMPK homotrimer prefers lower pH (especially pH6.8), and will eventually disappear at higher salt concentration (e.g. 500mM NaCl), no matter with (Fig. 1C) or without adding 1mM AMP (Fig. 1B). Taken together, both the pH and salt concentration would be the main factors that induce AMPK homotrimer formation rather than its functional cofactors, such as Mg^2+^ or AMP. In other word, local electrostatic interaction should play a critical role in AMPK homotrimer formation and stabilization.

### Structural comparability of AMPK with or without Mg^2+^

**Figure 2:**
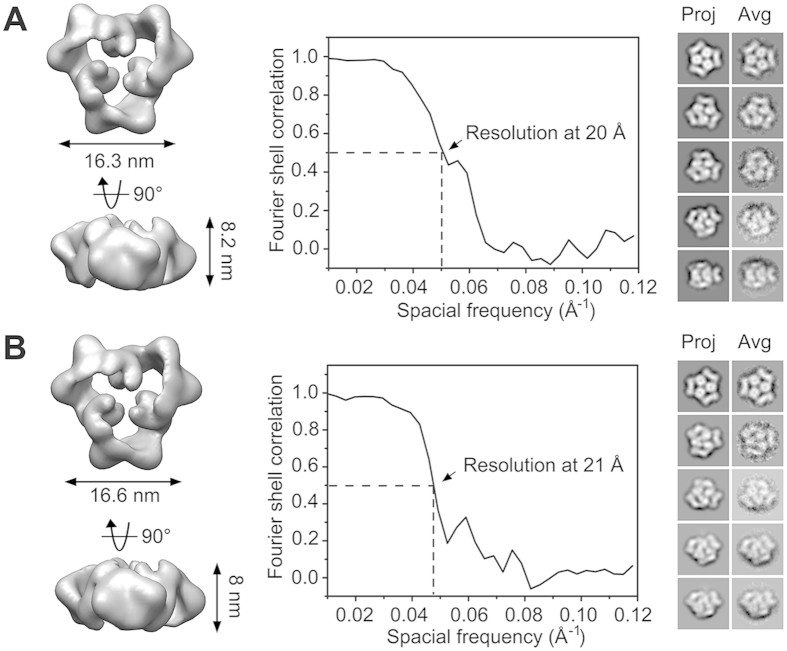
Three dimensional reconstruction of AMPK trimer in Mg^2+^-free buffer. The refined 3D model of AMPK (**A**) and AMP-bound AMPK (**B**) are shown in the left panel. The middle and right panel represents the assessment of model quality in Fourier Shell Correlation curve and projection-class average map comparison, respectively.

**Figure 3:**
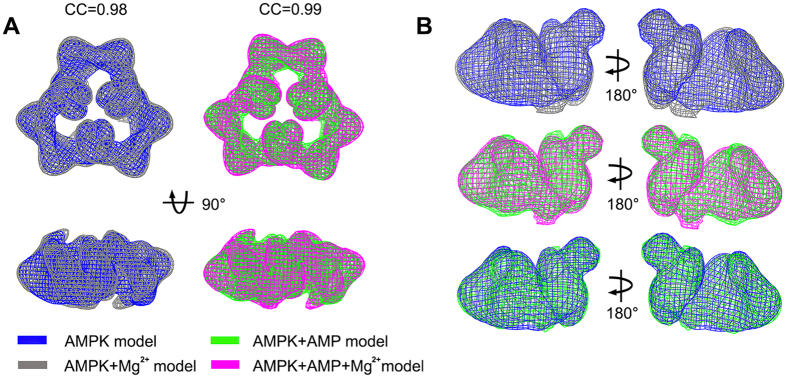
Comparison of AMPK and AMP-bound AMPK models with or without Mg^2+^. (**A**) Comparison of AMPK homotrimer model prepared in the present (colored in grey, EM accession code: EMD-1897) and absent (colored in blue) of Mg^2+^, and the AMP-bound AMPK homotrimer model with (shown in magenta, EM accession code: EMD-1899) or without (shown in green) Mg^2+^, the cross-correlation coefficient between each model pair shows on the above, which were generated from Chimera. (**B**) Comparison of three model pairs of monomers segmented from individual reconstruction.

Three-dimensional reconstructions were obtained from AMPK prepared in Mg^2+^-free buffer in the present and absent of AMP at a resolution of 20Å and 21Å with the FSC_0.5_ criterion, respectively (Fig. 2). It shows high similarity at both the 2D class average and 3D structural levels of these two samples, and they represented high cross-correlation with the previous models^20^ in Mg^2+^-containing buffer (Fig. 3A). Monomers segmented from those corresponding homotrimers are comparable with each other as well (Fig. 3B). Subtle differences between these models are mainly found at the fringe regions, presumably due to the relocation of flexible sequences caused by intra- or inter-molecular interaction. Therefore, the consistent shape of all the models generated with or without adding different cofactors indicates that neither Mg^2+^ nor AMP contributes to architectural determination of AMPK formed within a homotrimer, especially the location of KD or CBM.

### Architectural differences between EM model and crystal structure

**Figure 4:**
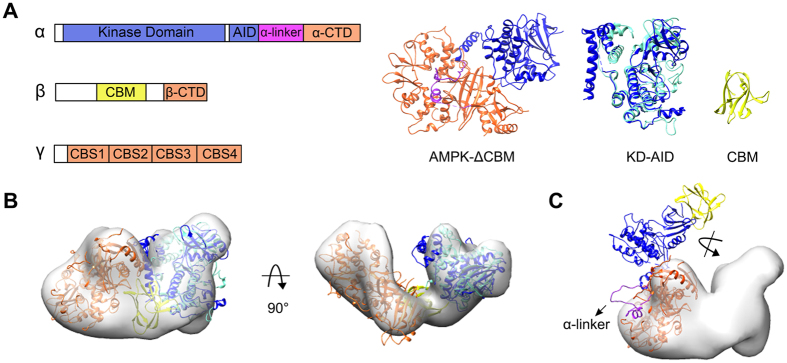
Schematic diagram of architectural determination. (**A**) Crystal structure of AMPK-ΔCBM (PDB code: 4CFH) and segmented KD-AID (AID superimposed with intact rat-α1-AID, PDB code: 4F2L, both colored in dark blue, together superimposed with previously used structure from *S. pombe,* PDB code: 3H4J, colored in cyan). CBM is from 1Z0M (PDB code, colored in yellow). (**B**) Representation of rigid-body docking of the individual KD-AID (including both from 4CFH and 3H4J), CBM and regulatory core complex (colored in orange) into AMPK monomer EM model based on previous proposed architecture. (**C**) Rigid-body docking of 4CFH overall structure into the same EM model. The location of CBM is aligned according to 4CFF (PDB code). All the structures are hidden of binding ligands.

Browsing the component information of those almost full-length crystallized AMPK complexes (Supplementary Table S1), two structures with PDB code 4CFH (rat-α1 human-β2 rat-γ1)^9^ and 4REW (human α1β2γ1)^5^ are in most close proximity to ours, in non-phosphorylation and AMP-bound state, except for a potent kinase inhibitor staurosporine binding to the KD and lacking two helices of AID as well as the entire CBM. When superimposed on each other, noticeable conformational difference of them mainly existed in the region between KD and α-CTD, which primarily includes AID and α-linker (Supplementary Fig. S1). According to the architecture revealed in the crystal structure of 4CFH (Fig. 4A), the distinct characteristic compared to our previously proposed model is about the orientation of KD-AID relative to the regulation core complex (Fig. 4B). If we docked the segmented KD-AID into our monomer EM density map separated from the homotrimer, it would require an anti-clockwise rotation and a shift against the CBS3-αRIM2 binding side (Fig. 4C). Compared with the situation that in our proposed model the well-structured AID is adjacent to the KD hinge region hence away from γ subunit, the incompletely structured AID in the crystalline AMPK is close to both KD and γ subunit and located in between them. However, it appears different orientation on the basis of the already structured α3-helix (Supplementary Fig. S1). Furthermore, when we superimposed the single KD-AID (human α1) crystal structure (PDB code: 4RED)^5^ upon either of the above constructs, AID rendered in an opposite direction hence conflicted with the trend of α-linker and occupied its position, even interrupted with the α-RIMs-γ-subunit interaction interface (Supplementary Fig. S2). It implies that it’s possible for the KD-AID inhibitor complex moving away from the regulation core complex as docked in the EM model. Besides, the overall α-linker (rat α1 a.a. 336–395, maximal ~20 nm long) connecting AID and α-CTD is largely disordered and partially unstructured^5^,^9^,^21^,^26^, providing a potential to the plasticity of location and orientation of KD-AID as rendered in the EM model.

While taking an insight into the phosphorylated crystal structures bound with AMP, which is considered as an active state, the α-RIM2 motif resolved tightly binding to CBS-3/AMP, meanwhile the α-RIM1 interacted with CBS-2 site^4^, leading the AID more close to the scaffold region, thereby releasing its inhibition to the KD. The contact area between αRIM motifs and γ subunit, which is formed predominantly by electrostatic and hydrophobic interactions as well as hydrogen bond^4^,^6^,^7^ is about 600 Å^2 ^9,26, indicating a modest interaction that may be disrupted under certain conditions such as competed with ATP binding, thus results in a conformational change and recovers the inhibition of KD by AID. Moreover, KD interacts with the regulatory core mainly through its activation loop forming hydrogen bond with β-CTD, which produces a contact area of ~500 Å^2 ^9,26. This relatively weak interaction would not be rather stable to maintain a conformation in some state. As shown in the superposition of 4RER (phosphorylated) and 4RED (unphosphorylated) in which the activation loop is unstructured, the confliction between AID and γ subunit (Supplementary Fig. S2) implied a probably different orientation and entire movement of KD-AID. Additionally, KD was involved in an inter-molecular interaction in crystalline AMPK^5^,^9^,^21^,^26^. For example, the buried surface area between KD N-lobe and the γ subunit of neighboring molecule in a unit cell is ~600 Å^2^. This successful crystal packing may be partially caused by electrostatic interaction between the positively charged KD N-lobe, and local negatively charged γ subunit (Supplementary Fig. S3). Consistently, tight interaction interface also formed according to the EM model, however in a different “end-to-end” circle mode^20^. Regardlessly, this indispensable interaction may also play a role in restricting the orientation of KD. Taking all these into account, the orientation of KD in the crystal structure is partially stabilized and restricted by multiple intra- and inter-molecular interactions, which in turn depends on different functional states.

Reviewing the architectural differences resolved by the two technologies, the location of CBM is another point. We docked this tightly structured globular domain into a solid density close to the scaffold region in between γ subunit and KD-AID (Fig. 4B), and the location is also confirmed by the comparison of CBM-truncated monomer with the one segmented from homotrimer, therefor presumed that it may take a part in the modulation of AMPK activity through interacting with the scaffold region^20^. However, as constructed in the phosphorylated and AMP bound crystal structures, it appeared more adjacently to the N-lobe of KD (Fig. 4C), binding with either the synthetic activators, such as compounds A769662 and 991^9^, or the glycogen-mimic cyclodextrin^5^ so as to stabilize AMPK in an active conformation. Nevertheless, without β-Ser108 been phosphorylated^5^,^9^ (including its phosphomimetic mutation)^21^ as the prerequisite as well as the molecular activators binding, currently no report demonstrates a mammalian construct with CBM co-crystallized, indicating that the location of CBM could render some flexibility, especially in the inactive non-phosphorylation state. As shown in its homologous structure of the heterotrimer core of *S. cerevisiae* SNF1, the CBM interacted with γ subunit, neighboring the scaffold region^27^, which indicated a potential rigid domain movement. Besides, tracing the linker bridging CBM and β-CTD in the already resolved structures, it primarily comprises disordered loop and still unstructured in some portion, representing in differential connecting path. Considering all above, since the architectural model of AMPK determined by EM is unphosphorylated neither at α-Thr172 nor β-Ser108, and absent of any synthetic small molecules binding with the CBM to stabilize its interaction with KD, it could have a possibility to relocate CBM in a different way other than shown in the crystalline AMPK.

## Discussion

Mammalian AMPK is a complicated kinase complex, having intra- and inter-molecular interaction between functional domains of each subunit. In spite of showing consistent characteristics such as KD-AID is adjacent to the scaffold region not the regulation core complex, AID is located behind KD hinge to inhibit its activity until α-Thr172 is phosphorylated, architectural analyses based on the structures generated from EM and X-ray crystallography have revealed its structural flexibility. AMPK appeared as multidispersed particles in solution, with monomers coexisting with certain proportional homo-oligomers^20^,^28^,^29^. This characterization was also presented in our EM observation whereas prevented it from being eligibly reconstructed by EM technology. After screened with combination of varied pH and salt concentration, we figured out an optimum condition (pH6.8 and 15 mM NaCl) for AMPK homotrimer forming and reconstructed its overall structure subsequently. On the basis of these stably existed homotrimers, we concluded that pH value is a key factor affecting AMPK homotrimer formation in solution while salt concentration is less effective, which further implied that electrostatic interactions should be crucial in mediating AMPK homotrimer formation. This finding is in accordance with our EM model, in which the α-KD N-lobe of each AMPK protomer enriching positive charges tightly interacted with its neighboring protomer’s γ subunit through its negatively charged residues^20^. Probably this intermolecular interaction would restrict the orientation of α-KD and γ subunit in the AMPK homotrimer. Notably, analysis of unit cell of holo-AMPK crystal structure shows intermolecular interactions between KD N-lobe and γ subunit from the neighboring as well. In the EM model, AID bound to inhibit the unphosphorylated KD thus may also contribute to relocate its orientation. In conclusion, the inter-molecular interaction between protomers when forming homotrimer as well as the flexible linkers connecting those functional domains should play an important role in mediating KD movement rather than the AMPK cofactors or the phosphorylation states of α-Thr172 and β-Ser108.

Multisubunit proteins posses more complicated regulation mechanism thus varied conformations in the process of function. As a central energy sensor, AMPK is modulated by various upstream molecules and controls cellular metabolism through directly phosphorylates a wide range of substrates including various metabolism-related enzymes and/or their gene transcription involved proteins^3^,^30^,^31^,^32^,^33^. Given the complicated function and regulation mechanism of AMPK, it seems reasonable that AMPK should possess certain flexibilities to adapt to the broad range of interaction molecules. The structural plasticity of AMPK may explain the architectural differences between the crystal structure and the EM model, which are primarily conferred by the flexible subunit linker sequences. The crystal structure represents an AMP-activated conformation of AMPK, but whether this conformation is available in solution is unclear. Despite many efforts devoted, crystal structure of full-length AMPK is remain unresolved so far due to crystallization difficulty. In currently solved crystal structures of AMPK heterotrimer, flexible sequences are excluded more or less in favor of crystallization. Despite at relative low resolution, our EM model provides AMPK architecture in full-length. The EM structure shows a state of unphosphorylated AMPK in solution, which can be activated by AMP if phosphorylated by upstream kinases, indicating it may stands for a physiological available form. The conformational change between inactive and active AMPK still require further investigation of high-resolution structures.

## Methods

### AMPK preparation

Full-length rat AMPK (α1β1γ1) was prepared as previously described^20^,^34^. Briefly, the holoenzyme was inserted into a tricistronic vector and expressed in *Escherichia coli* BL21 (DE3) at 20 °C and sequentially purified by Ni-NTA resin (QIAGEN), ion exchange and gel filtration columns (Source-15Q/15S and Superdex-200, GE Healthcare). The purified protein were stored in aliquots at 80 °C. The structure and functional states of the samples were further analyzed by electrophoresis, enzyme activity assay and size exclusion chromatography. The α-Thr172 and β-Ser108 were unphosphorylated as verified by time-of-flight mass spectrometry analysis^20^.

### Data collection in Mg^2+^-free buffer

AMPK was directly diluted to ~0.2 mg/ml with buffer (20 mM Hepes, pH 6.8, 15 mM NaCl, 1 mM TCEP) or incubated with 1mM AMP at 18 °C for 1 h until negatively stained for 30 s with 1% uranyl acetate on the 300 mesh continuous carbon covered and glow discharged copper grids^20^. Micrographs were collected at a nominal magnification of 50,000× on a FEI Tecnai G^2^ electron microscope operating at 200 kV, with a total electron dose of ~22e^−^/Å^2^s.

### Single particle reconstruction

We performed Random Conical Tilt (RCT) reconstruction for homotrimeric AMPK data processing due to their preferred orientation as described in our early work^20^. Briefly, 4029 and 3729 tilt-pairs (0° and 45°) were picked for AMPK and AMPK + AMP samples respectively using SPIDER/WEB program^35^. The untilted particles were subjected to reference-free alignment and classified into ten groups for subsequent multi-reference alignment using SPIDER^35^,^36^ scripts and EMAN2 programs^37^. Individual initial model was generated from corresponding tilted data sets. High-quality models with cross-correlation coefficient better than 0.89 were merged to build a new volume, followed by projection-matching and further angular refinement. In the last refinement iteration, c3 symmetry was applied to generate the final model.

### Rigid-body docking

The reconstructed models of AMPK without Mg^2+^ were shown and analyzed by using UCSF Chimera^38^,^39^ with a contoured mass of 389 kDa assuming a protein density of 1.35 g/ml (0.81 Da/Å^3^). In order to compare with each other, all the models were Gaussian low-pass filtered to 21 Å according to the quality assessment by Fourier Shell Correlation (FSC), and the homotrimer structure was segmented into three monomers using ‘segment map’ tool in Chimera. Crystal structures of KD-AID (PDB code: 3H4J, chain B), CBM (PDB code: 1Z0M, chain A) and regulatory core complex (PDB code: 2V9J) were docked into AMPK EM density map using SITUS^40^ and Chimera according to our previously proposed AMPK architecture^20^.

### Electrostatic surface potential analysis

We performed software PMV^41^ that uses Adaptive Poisson Boltzmann Solver (APBS) to calculate electrostatic properties for all the related crystal structure files we mentioned in this work. We attempted different force field such as AMBER, CHARMM and PARSE to generate files by pdb2pqr package, and after we colored the surface map according the electrostatic potential, no significant difference was detected between them. We used Chimera for visualizing the electrostatic potential results, and colored positive potential in blue, while negative potential in red.

## Additional Information

**How to cite this article**: Ouyang, Y. *et al.* Architectural plasticity of AMPK revealed by electron microscopy and X-ray crystallography. *Sci. Rep.* **6**, 24191; doi: 10.1038/srep24191 (2016).
